# Effects of different types of vegetation cover on soil microorganisms and humus characteristics of soda-saline land in the Songnen Plain

**DOI:** 10.3389/fmicb.2023.1163444

**Published:** 2023-09-21

**Authors:** Liangliang Guo, Tibor Tóth, Fan Yang, Zhichun Wang

**Affiliations:** ^1^Northeast Institute of Geography and Agroecology, Chinese Academy of Sciences, Changchun, China; ^2^University of Chinese Academy of Sciences, Beijing, China; ^3^Centre for Agricultural Research, Institute for Soil Sciences, Budapest, Hungary

**Keywords:** vegetation restoration, physical properties of soil, soil humus carbon component, soda-saline soil, soil microorganism

## Abstract

**Introduction:**

In the soda-saline grasslands of the Songnen Plain, Jilin Province, China, the prohibition of grazing has led to significant changes in plant communities and soil properties. However, the intricate interplay between soil physical and chemical attributes, the soil microbial community, and their combined influence on soil humus composition remains poorly understood.

**Methods:**

Our study aimed to evaluate the impact of natural vegetation restoration on soil properties, microbial community diversity, and composition in the soda-saline soil region of the Songnen Plain. We conducted assessments of soil physical and chemical properties, analyzed community diversity, and composition at a soil depth range of 0–20 cm. The study covered soils with dominant soda-saline vegetation species, including *Suaeda glauca* Bunge, *Puccinellia chinampoensis* Ohwi, *Chloris virgata* Swarta, *Phragmites australis* (Clay.), *Leymus chinensis* (Trin.), and Tzvelev. We compared these vegetated soils to bare land devoid of any plants.

**Results:**

We found that soil organic content (SOC) in vegetation restoration areas was higher than in bare land, with SOC content varying between 3.64 and 11.15 g/kg in different vegetated areas. Notably, soil pH emerged as a pivotal factor, explaining 11.4% and 12.2% of the variance in soil bacteria and fungi, respectively. There were correlations between SOC content and the relative abundance of specific microbial groups, with Acidobacteria and Mortierella showing a positive correlation, while Actinobacteria, Gemmatimonadetes, and Ascomycota exhibited significant negative correlations with SOC.

**Discussion:**

The disparities in SOC composition and content among the soda-saline vegetation types were primarily attributed to variations in pH. Consequently, reducing soil pH is identified as a critical step in the process of vegetation restoration in soda-saline land. Prohibiting grazing has the potential to increase soda-saline SOC content and enhance microbial diversity, with *Leymus chinensis* and *Phragmites australis* showing particularly promising results in terms of higher SOC carbon content and microbial diversity.

## 1. Introduction

**Figure 1:**
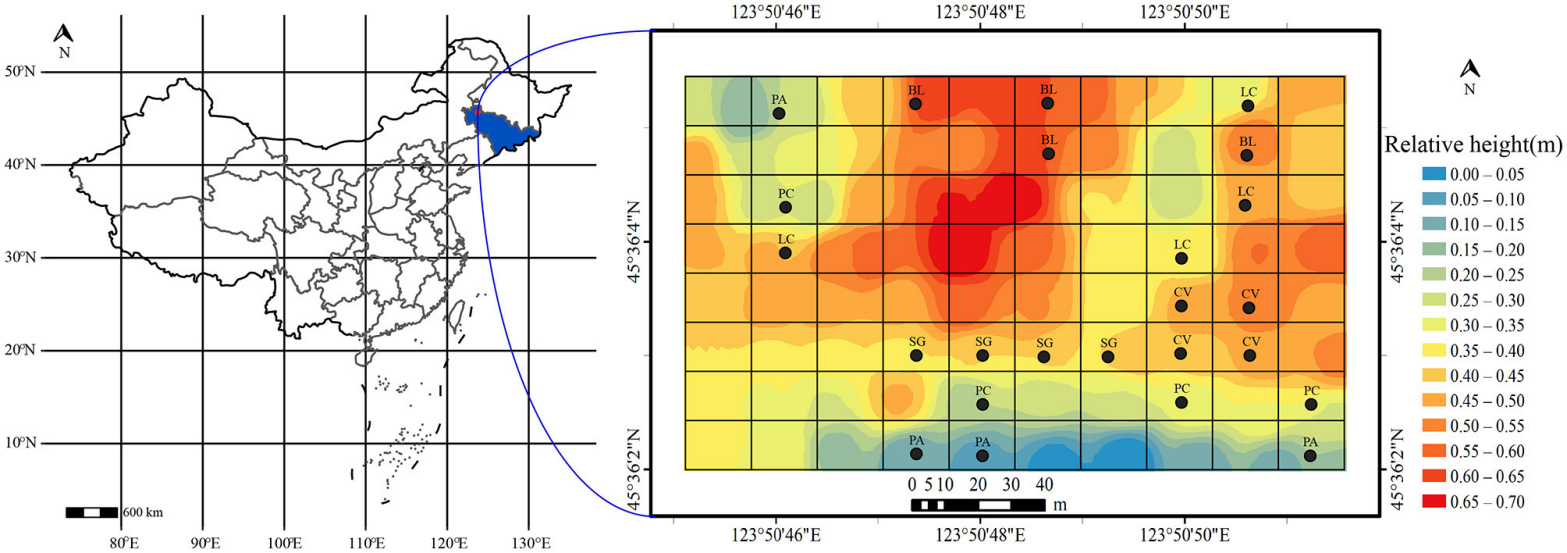
The location of the experiment and the distribution of sampling squares. BL, bare land without vegetation on the surface; CV, *Chloris virgata*; LC, *Leymus chinensis*; PA, *Phragmites australis*; PC, *Puccinellia chinampoensis*; SG, *Suaeda glauca*.

The western part of the Songnen Plain is located in the northeast of China. It is one of the three major soda-saline soil regions in the world (Yang et al., 2016), with an area of 3.42 × 10^6^ ha (Wang et al., 2003). NaHCO_3_ and Na_2_CO_3_ are the main salts there, with soil pH mostly above 8.5, often ranging between 9.0 and 10.5, which is the characteristic pH value of severe soda-saline land (Chi et al., 2011). In the past, due to long-term salinization and human disturbances, the grassland in this region has suffered severe degradation, leading to a decline in soil productivity and almost no surface vegetation (Zhao et al., 2022). To restore the damaged ecosystem, a no-grazing policy has been implemented since 2002, allowing natural vegetation to regrow and restore soil quality and functionality. After years of regeneration, there has been an increase in vegetation cover, and a relatively stable natural ecology has been established through long-term succession. Additionally, diverse plant communities have emerged due to variations in microtopography, habitats, and degrees of degradation. Plants such as *Suaeda salsa, Puccinellia chinampoensis, Phragmites australis, Chloris virgata*, and *Leymus chinensis* exhibit a patchy distribution across plots with different levels of salinization (Figure 1).

The status and movement of soil water and salt determine the formation of different vegetation communities, while the change in soil salinity is influenced by various factors, such as climate conditions, surface and subsurface water, vegetation types, and field management. Vegetation growth also contributes to the accumulation of soil organic matter (SOM) (Mahmood et al., 1994). SOM is a mixture of special polymer compounds formed by animal and plant residues in the soil under the action of soil organisms (Kögel-Knabner, 2002). The main active components of SOM are humic substances (HS), which are produced by chemical activities in natural environments, including aquatic ecosystems, soils, and sediments, and have a major impact on biogeochemical processes (DiDonato et al., 2016; Zaiets and Poch, 2016). HS can be further classified into the groups of humic acid (HA), fulvic acid (FA), and humin (HM) (Kou et al., 2022). HA and FA are the most active components in soil HS and play important roles in enhancing soil aggregation, maintaining soil fertility, increasing soil buffering capacity, and regulating pH (Lehmann and Kleber, 2015). Different soil types have different HA/FA ratios (Klučáková, 2018; Ampong et al., 2022). At the same time, this ratio is related to soil fertility (Kononova, 2013). SOM belongs to the soil solid phase, and most of it combines with minerals to form organomineral complexes or microaggregates of different sizes. High soil salinity has a certain impact on the structure, composition, and characteristics of soil humus, which are closely related to the properties and fertility of the soil. Therefore, the study of the correlation between salinity and humus composition in soda-saline soil is of great theoretical and practical significance for the formation, regeneration, and decomposition of humus in soda-saline soil, revealing the migration and transmission mechanisms of substances between saline soil and vegetation types.

The soil microbiome plays a pivotal role in soil ecological processes (Hartmann and Six, 2022). Soil electrical conductivity and soil sodium ion content are the primary factors influencing the structure and composition of soil bacterial communities in soda-saline land (Wang S. et al., 2020). Notably, soil salinity not only significantly diminishes bacterial richness but also affects bacterial community composition (Guan et al., 2021). In a study by Chang et al. (2022), it was revealed that soda-saline soils covered with natural vegetation have positive effects on soil physicochemical properties and prokaryotic communities, as opposed to highly saline bare sodic spots. Generally, diverse plant communities create distinct soil environments, leading to shifts in soil microbial communities (Wang X. et al., 2020; Qiu et al., 2022). These alterations in microbial diversity and community composition undoubtedly influence the interplay between the microbiome and nutrient cycling, subsequently affecting plant growth in saline soils.

Conversely, certain fungi, such as mycorrhizae, possess the capacity to assist plants in resource acquisition and high salinity tolerance within stressful settings (Wang et al., 2019). Moreover, fungi collaborate with bacteria to uphold the stability of soil microbial networks and contribute to ecosystem functionalities (Li et al., 2022). Revegetation affects land productivity and the hydrological cycle and changes the structure and function of soil microbial communities and humus. Soil microorganisms and humus are the main components of soil organic carbon, and they play an important role in the soil carbon cycle and global carbon balance. However, there is still a lack of systematic research on the changes and mechanisms of soil microorganisms and humus in different stages of vegetation degradation in the Songnen Plain.

Based on the aforementioned considerations, we postulate that soils encompassing various plant communities exhibit distinct organic matter components and diminished salt stress. Consequently, the soil within diverse plant communities harbors greater microbial diversity functionality than the highly saline and alkaline barren soil patches. To scrutinize this conjecture, we studied the diversity and composition of both bacterial and fungal communities in soils beneath five halophytic communities within a soda-saline ecosystem. Our analysis encompassed the measurements of soil pH, electrical conductivity, salinity, organic matter composition, salt stress, and microbial functions. We quantified the ratio of soil bacteria to archaea and assessed fungal diversity and composition using advanced high-throughput sequencing techniques. Notably, we identified indicator taxa that exhibited a significant association with specific habitats. Primarily, the present study aimed to explore the impact of distinct vegetation types on organic matter composition and the interrelationship of microorganisms within soda-saline soils.

## 2. Materials and methods

### 2.1. Site description

The study area is situated in the Da'an Sodic Land Experimental Ecological Station (45°53′-47°8′N, 123°45′-124°42′E), located in the west of the Songnen Plain. It has an average elevation of 152 m and an annual average temperature of 3.6–4.4°C. The monthly average temperature stays above 0°C from April to October. Soda-saline meadow soil is the dominant soil type. Due to grazing before 2002, the experimental area has been exposed to heavy salinity for a long time, characterized by severe grassland degradation and low vegetation cover. Since early 2002, grazing has been prohibited on grasslands, leading to a subsequent increase in vegetation coverage. Over the course of several years, through long-term succession, a relatively stable and natural ecological system has been established. Additionally, diverse plant communities arose as a result of variations in minor topography, habitat, and degree of degradation.

We divided the study site into 80 plots of 15 × 15 m each, which were randomly assigned to one of five treatments (Figure 1). The study site was 120 m wide (1.8 × 10^4^ m^2^). The following six vegetation communities were distinguished and sampled, I. Bare land (BL) without vegetation on the surface; II. *Chloris virgata* (CV); III. *Leymus chinensis* (LC); IV. *Phragmites australis* (PA); V. *Puccinellia chinampoensis* (PC); VI. *Suaeda glauca* (SG); see details in Supplementary Table S1.

### 2.2. Soil sampling

Soil samples were collected in September 2022, when vegetation matures. Plant residues were removed prior to sampling to avoid aboveground disturbance. Soil samples were collected using the five-point sampling method. Five cylindrical soil samples were randomly drilled in each square with a diameter of 5 cm and a depth of 20 cm. They were broken up and thoroughly mixed into a composite soil sample, with each square replicated four times. Finally, 24 mixed soil samples (6 vegetation variants × 4 replicates) were obtained. Shoots, roots, and gravel were manually removed from the soil samples. Soil samples were then passed through a 2-mm sieve and divided into three equal aliquots. The first one was air-dried at room temperature and used for physicochemical analysis; the second sample was immediately frozen at −80°C and used for microbiological analysis; and the third sample was also air-dried at room temperature but kept in a dark and dry place for backup purposes.

### 2.3. Soil analysis

Soil bulk density was measured using the ring knife method. The analysis and determination methods for soil humus and optical properties were adopted from the study by Dou (2010). Specific experimental operations were as follows: 5.00 g of oil passed through a 0.25-millimeter sieve was weighed into a low-speed centrifuge tube; 30 ml of distilled water was added and stirred evenly with a glass rod; the tube was then placed on a constant temperature water bath oscillator (70 ± 2°C, 145 r/min) for 1 h and centrifuged at a low speed of 3,500 r/min for 15 min. The supernatant was filtered into a 50-ml volumetric bottle with a quantitative filter paper, and the residue was washed once with 20 ml distilled water to obtain WSS (water-extracted organic matter). Moreover, 30 ml of lye mixed from 0.1 mol/L NaOH and Na_4_P_2_O_7_ was added to the residue in the centrifugation tube, mixed evenly with a glass rod, and then extracted for 1 h by shaking on a constant temperature water bath oscillator (70 ± 2°C, 145 r/min). After the removal, it was centrifuged at 3,500 r/min for 15 min, filtered with a quantitative filter paper into a 50-ml volumetric bottle, and the residue was washed with 20-ml mixed lye, filtered into the volumetric bottle, and filled up with distilled water to a constant volume. The soluble solution is HE (extractable humus substance). Then, 30 ml of the HE solution was poured into a 50-ml Erlenmeyer flask, and 0.5 mol/L of H_2_SO_4_ solution was added to adjust the pH to 1.0–1.5. The acidified solution was incubated in a water bath at 60–70°C for 2 h and left to rest overnight. The following day, the solution was filtered with a quantitative filter paper, the precipitate was HA (humic acid), and the solution was FA (fulvic acid). The HA precipitate was washed three times with a 0.05-mol/L H_2_SO_4_ solution, and the solution was discarded. Finally, the HA precipitate was dissolved in a warm (60°C) 0.05-mol/L NaOH solution in a 50-ml volumetric bottle and finally dissolved in a 0.05-mol/L NaOH solution. The residue in the centrifuge tube was humin (HM), which was washed in distilled water three times. After centrifugation, the precipitate was dried, finely ground, and screened for determination. FA and HA were measured using a Shimadzu TOC analyzer (TOC-L, CPH). Analysis of SOM and HM was performed using the K_2_CrO_7_-H_2_SO_4_ oxidation method (Walkley and Black, 1934).

The PQ value is an indicator of the degree of humification in the soil. It is commonly used to assess the maturity and stability of soil organic matter. The PQ value is calculated by dividing the content of humic acid (HA) by the sum of humic acid (HA) and fulvic acid (FA). A higher PQ value indicates a higher proportion of humic acid in the organic matter, indicating a higher degree of humification. The changes in PQ value can reflect the processes of organic matter formation and decomposition in the soil and the influence of environmental factors such as soil texture and vegetation type on organic matter. The calculation formula is as follows:


(1) $$\begin{array}{l} PQ=\frac{HA}{HA+FA} \end{array}$$

A 1:5 soil-water ratio was used to extract and analyze water-soluble salts. pH, EC, Na^+^, K^+^, Ca^2+^, Mg^2+^, ${\text{CO}}_{3}^{2-}$, ${\text{HCO}}_{3}^{-}$, Cl^−^, ${\text{SO}}_{4}^{2-}$ and other major ion concentrations were determined. A conductivity meter was used to measure EC_1:5_, and a pH meter was used to measure soil pH_1:5_. Na^+^, K^+^, Ca^2+^, and Mg^2+^ ion concentrations were determined using atomic absorption spectrophotometry, and ${\text{CO}}_{3}^{2-}$ and ${\text{HCO}}_{3}^{-}$ were determined using double indicator neutralization titration. Cl^−^ ions were determined using silver nitrate titration and ${\text{SO}}_{4}^{2-}$ ions using barium sulfate turbidimetry.

The soil sodium adsorption ratio (SAR) is an indicator that measures the relative proportion between the content of sodium ion and the content of other ions, such as calcium and magnesium, in the soil. It can be used to assess the degree of soil salinization and alkalization. SAR is calculated using Equation 2 (Chi et al., 2011):


(2) $$\begin{array}{l} SAR=\frac{\left[Na^{+}\right]}{\sqrt{\left(\left[Ca^{2+}\right]+\left[Mg^{2+}\right]\right)/2}}, \end{array}$$

where [Na^+^], [Ca^2+^], and [Mg^2+^] are the corresponding ion concentrations in mmol_c_/L, and the unit of SAR is (mmol_c_/L).

Total alkalinity was calculated using the following formula:


(3) $$\text{Total }{\text{alkalinity=CO}}_{3}^{2-}{\text{+HCO}}_{3}^{-}({\text{mmol}}_{\text{c}}\text{/L})$$

Mean weight diameter (MWD) stands for mean weight diameter, which is a measure of soil aggregate stability. Soil aggregates are groups of soil particles that are bound together by various forces. Soil aggregate stability indicates how well the aggregates resist breaking apart when exposed to external forces, such as water or wind erosion. Soil aggregate stability is important for soil structure, water infiltration, nutrient cycling, and plant growth. The wet sieving method was used to divide soil aggregates into four size fractions: (i) macroaggregates (>2.0 mm), (ii) small aggregates (0.25–2.0 mm), (iii) microaggregates (0.053–0.25 mm), and (iv) silt and clay (<0.053 mm). Approximately 100 g of air-dried soil moistened to field capacity after soaking was immersed in water over a sieve (2 mm, 1 mm, 0.25 mm, and 0.053 mm), oscillating at a frequency of 30 times per minute for 2 min with a vertical amplitude of 3 cm. Soil aggregates on the sieve were collected, dried at 50°C for 3 days, and weighed. Material passing through the 0.053-mm sieve was not collected but calculated by subtracting the remaining weight from the total 100 g.

The MWD was calculated using Equation 4:


(4) $$\begin{array}{l} MWD=\overset{n}{\underset{i=1}{\sum }}Xi\;Wi, \end{array}$$

where Xi is the average diameter (mm) of sieved aggregates in any particle size range and Wi is the weight percentage (%) of aggregates in any particle size range, where i = 1, 2, …, 4 represent the aggregate size classes such as >2.0 mm, 0.25–2.0 mm, 0.053–0.25 mm, and <0.053 mm, respectively (Kemper and Rosenau, 1986).

### 2.4. Soil DNA extraction, PCR amplification, and Illumina sequencing

Nucleic acid was extracted using the OMEGA Soil DNA Kit (D5625-01) (Omega Bio-Tek, Norcross, GA, USA). For the extracted DNA, 0.8% agarose gel electrophoresis was carried out to determine the molecular size, and a UV spectrophotometer was used to quantify the DNA. In this analysis, the 16S rRNA gene was amplified by PCR using the primer pairs 341F (5′-barcode + CCTACGGGNGGCWGCAC-3′), 806R (5′-GGACTACHVGGGTWTCTAAT-3′). The ITS2 rDNA gene was amplified by PCR using the primer pairs ITS3-KYO2 (GATGAAGAACGYAGYRAA) and ITS4 (TCCTCCGCTTATTGATATGC). Later, 2 × 250-bp paired-end sequencing was performed on the Illumina NovaSeq machine using the NovaSeq 6000 SP Reagent Kit (500 cycles).

We implemented a complete pipeline using the DADA2 R package (Callahan et al., 2016) (version 1.14) to convert pair-ended fastq files from the sequencer to merged, denoised, chimera-free, and inferred sample sequences. Paired-end denoised reads were merged into raw Amplifier Sequence Variant (ASV) with a minimum overlap of 12 bp. Chimera sequences were identified and deleted by the UCHIME algorithm (Edgar et al., 2011). After the removal of chimeras, the denoised, chimera-free ASV sequences and their abundances were output. Finally, a naive Bayesian model with a confidence threshold value of 0.8 was used to classify the representative ASV sequences into different organisms using an RDP classifier (Wang et al., 2007) (version 2.2) based on the SILVA database (Pruesse et al., 2007) (version138.1) or the ITS2 UNITE (Unified System for the DNA-based fungal species linked to the classification) database (Ankenbrand et al., 2015) (version update_2015). We excluded chloroplasts, mitochondria, and rare ASVs (relative abundance <0.005%) of bacteria and archaea.

### 2.5. Statistical analysis

Differences in the physicochemical properties and the microbial characteristics of soil under different plant communities were compared using a one-way analysis of variance and LSD tests. To meet the statistical premise of homoscedasticity of data, the data were passed through Levene's test based on the raw or log-transformed data before the ANOVA and LSD tests. The correlation between them was analyzed using Pearson correlation analysis. The R (4.1.3) “linkET” package (https://github.com/Hy4m/linkET) was used to plot heatmaps. The R “vegan” package was used to perform principal coordinate analysis (PCoA) to reveal differences in soil bacterial communities under different communities, and the Mantel test and redundancy analysis (RDA) were performed to investigate the relationship between environmental factors and bacterial and fungi community structure. Permutational multivariate analysis of variance (PERMANOVA) was performed on the Bray-Curtis distance using Adonis from the vegan package (https://github.com/vegandevs/vegan). Variance partitioning was analyzed using the varpart function from the vegan package to assess the relative effects of different plant community types, soil properties, and electrical conductivity on changes in community composition. Indicator species analysis was performed using the indicspecies package to identify taxa significantly associated with a given habitat at the ASV level (https://github.com/cran/indicspecies), and the significance of the association was evaluated at a false discovery rate (FDR) with a corrected *p*-value of <0.05 (Benjamini and Hochberg, 1995).

## 3. Results

### 3.1. Soil properties of different plant communities

Soil physical and chemical properties under the investigated plant communities differed significantly (Table 1). The pH, EC, SAR, total alkalinity, and bulk density of LC and PA were significantly lower than those of other plant communities. LC and PA significantly increased the MWD of the soil. The soil organic carbon content under different vegetation communities varied from 3.64 to 11.15 g/kg, and the soil organic carbon content of the LC community was the largest. The trend of water-soluble organic carbon (WSS) content differed from that of organic carbon, and the WSS content was higher in PA and BL. The largest constituent of organic matter was humin, and its content under LC was the highest. However, the humin content under PC was significantly higher than that under PA. Fulvic acid and humic acid contents were significantly different in under-investigated plant communities, and their content under LC was the highest. The PQ ratio, which indicates the degree of humification of soil organic matter, ranged from 0.53 to 0.63 among different plant communities. LC had the highest PQ ratio (0.63), indicating a higher degree of humification and a higher quality of humus, while PA had the lowest PQ ratio (0.53), indicating a lower degree of humification and a lower quality of humus.

**Table 1:** Soil chemical properties under different plant communities.

	**BL**	**CV**	**SG**	**PC**	**PA**	**LC**
WSS (g·kg^−1^)	0.17 ± 0.02^b^	0.12 ± 0.01^cd^	0.11 ± 0^d^	0.11 ± 0.01^d^	0.23 ± 0.02^a^	0.13 ± 0.02^c^
FA (g·kg^−1^)	0.19 ± 0.02^f^	0.52 ± 0^c^	0.44 ± 0^d^	0.27 ± 0.03^e^	1.11 ± 0.06^b^	1.25 ± 0.04^a^
HA (g·kg^−1^)	0.26 ± 0.02^e^	0.65 ± 0.01^c^	0.65 ± 0.02^c^	0.36 ± 0.02^d^	1.26 ± 0.17^b^	2.13 ± 0.05^a^
HM (g·kg^−1^)	2.98 ± 0.04^e^	4.42 ± 0.23^d^	5.68 ± 0.19^c^	6.67 ± 0.18^b^	5.71 ± 0.11^c^	7.76 ± 0.19^a^
SOC (g·kg^−1^)	3.64 ± 0.02^e^	5.97 ± 0.06^d^	7.04 ± 0.02^c^	7.15 ± 0.05^c^	8.24 ± 0.2^b^	11.15 ± 0.29^a^
PQ	0.57 ± 0.04^bc^	0.56 ± 0.01^bc^	0.6 ± 0.01^ab^	0.57 ± 0.01^bc^	0.53 ± 0.05^c^	0.63 ± 0^a^
pH	10.65 ± 0.05^a^	10.42 ± 0.08^b^	10.63 ± 0.03^ab^	10.53 ± 0.06^ab^	9.51 ± 0.13^c^	9.39 ± 0.27^c^
EC_1:5_ (dS·m^−1^)	4.86 ± 0.84^a^	0.87 ± 0.14^c^	2.34 ± 0.46^b^	1.19 ± 0.06^c^	0.18 ± 0.04^d^	0.18 ± 0.07^d^
BD (g·cm^−3^)	1.55 ± 0.02^a^	1.48 ± 0.04b	1.47 ± 0.03^bc^	1.41 ± 0.05^c^	1.33 ± 0.04^d^	1.31 ± 0.05^d^
Total alkalinity (mmol_c_L^−1^)	32.2 ± 8.84^a^	6.85 ± 0.66^cd^	17 ± 2.4^b^	9.85 ± 1.09^c^	2.3 ± 0.35^d^	2.5 ± 0.35d
SAR (mmol_c_L^−1^)	28.5 ± 6.34^a^	6.34 ± 1.37^cd^	17.43 ± 2.23^b^	8.19 ± 0.66^c^	2.23 ± 0.63^d^	2.39 ± 0.92^d^
MWD	0.26 ± 0.01^cd^	0.46 ± 0.03^b^	0.19 ± 0.06^d^	0.31 ± 0.05^c^	0.61 ± 0.06^a^	0.64 ± 0.04^a^
Soil moisture content (%)	11.75 ± 2.22^c^	14.00 ± 1.82^c^	11.89 ± 1.44^c^	26.01 ± 2.90^a^	19.11 ± 1.19^b^	23.45 ± 1.08^a^

Values are the mean ± standard error (*n* = 4); the different letters in the same row indicate significant differences (*p* < 0.05) at different restoration types via the LSD test.

BL, bare land without vegetation on the surface; CV, *Chloris virgata*; LC, *Leymus chinensis*; PA, *Phragmites australis*; PC, *Puccinellia chinampoensis*; SG, *Suaeda glauca*; WSS, water extracted organic matter; FA, fulvic acid; HA, humic acid; HM, humin; SOC, soil organic carbon; EC, electrical conductivity; BD, bulk density; SAR, sodium adsorption ratio; and MWD, mean weight diameter.

### 3.2. Soil bacterial community composition and diversity

**Figure 2:**
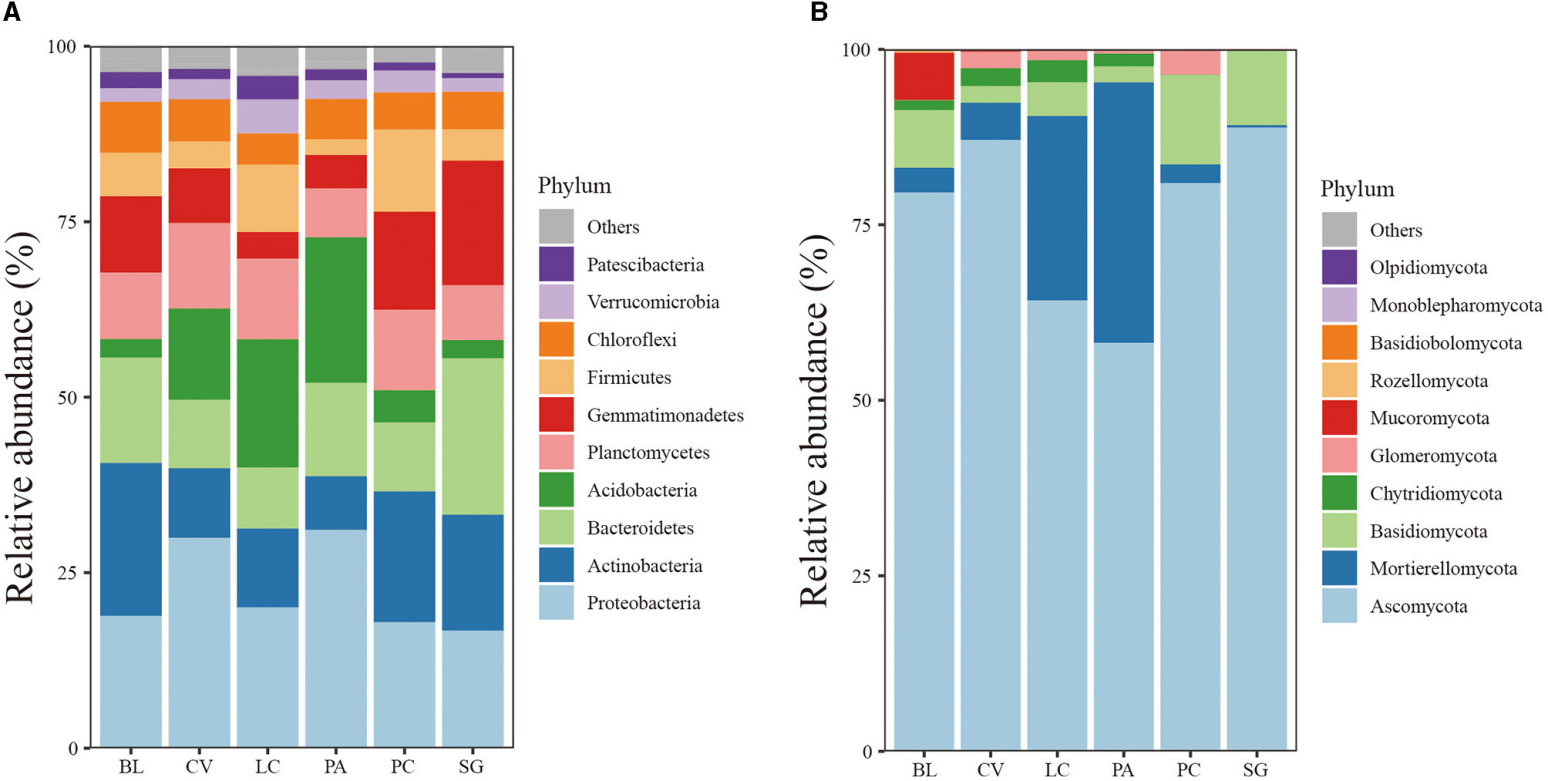
Relative abundances of the soil bacterial **(A)** and fungal communities **(B)** at the phylum level under the investigated plant communities.

There are 36 phyla of bacterial communities in the soil of different plant communities, mainly composed of Proteobacteria (20.2%), Actinobacteria (12.84%), Bacteroidetes (11.96%), Acidobacteria (9.16%), Gemmatimonadetes (8.88%), Planctomycetes (8.87%), Firmicutes (5.73%), and Chloroflexi (5.15%). Eleven phyla of fungi were identified, with the most prevalent being Ascomycota (61.77%), Mortierellomycota (10.69%), and Basidiomycota (5.26%). Among bacteria, the relative abundance of Actinobacteria in LC, CV, and PA was significantly decreased and Acidobacteria was significantly increased in LC and PA community soils (Figure 2). In addition, Verrucomicrobia and Patescibacteria were increased in LC soil. The abundance of Bacteroidetes and Gemmatimonadetes was highest in SG soil. Most of the fungi belonged to Ascomycota, especially in SG and CV community soil samples. PA and LC communities promoted the abundance of Mortierellomycota, and BL provided the highest abundance of Mucoromycota out of all communities.

**Figure 3:**
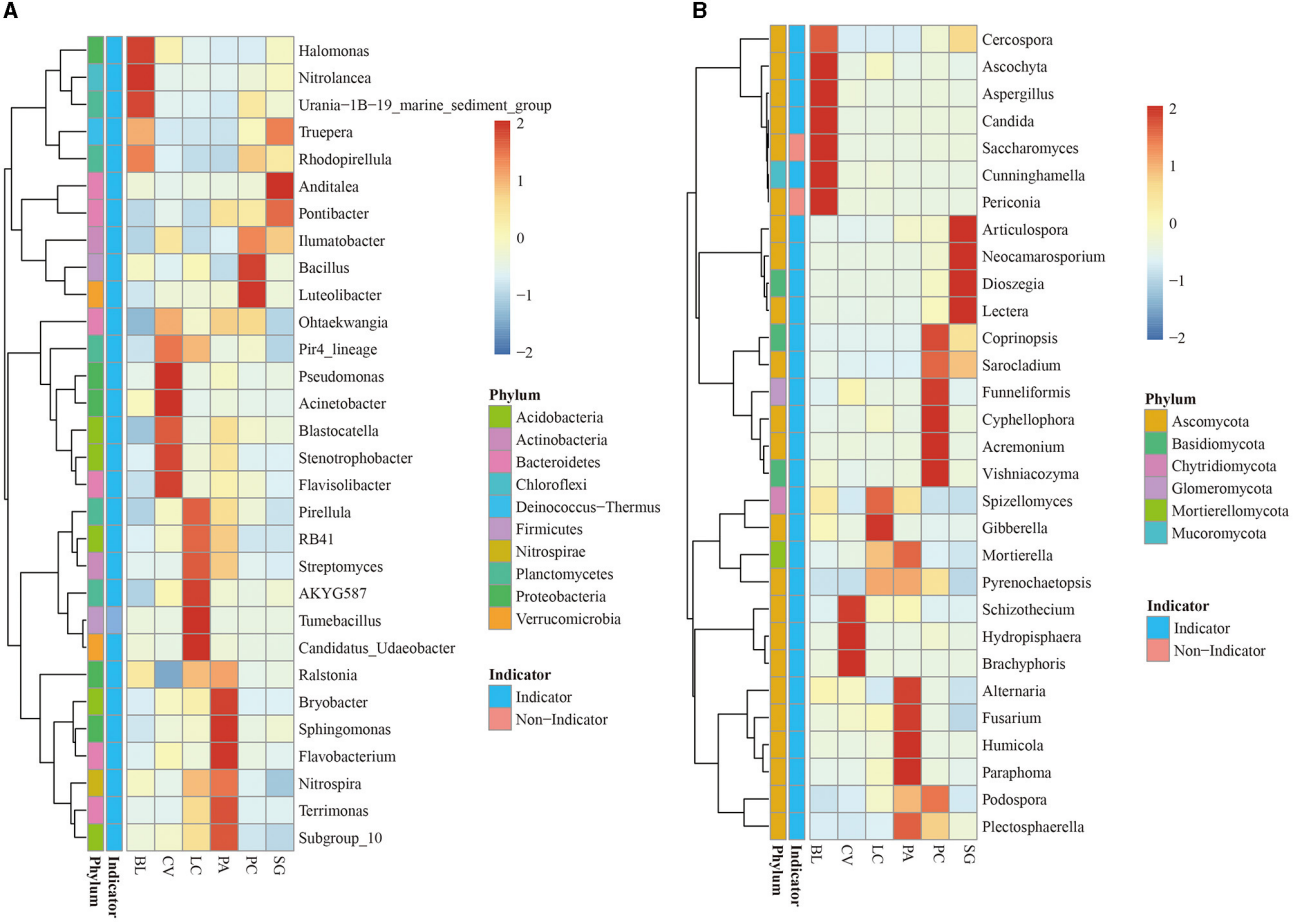
Distribution of the top 30 most abundant taxa (genus), their relative abundance, and indicator roles in the bacterial **(A)** and fungal **(B)** communities.

The 30 genera of bacterial and fungal communities were analyzed further to compare their relative abundance, indicator species status, and ecological functions in different plant communities. The relative abundance of the 30 most abundant genera accounted for 20.61% and 37.19% of bacterial and fungal abundance, respectively (Figure 3). Most of these genera (29 for bacteria and 29 for fungi) were also identified as indicator taxa. Abundant bacteria came from Proteobacteria, Planctomonas, Bacteroidetes, and Acidobacteria, belonging to *Halomonas, Nitrolancea*, and *Urania-1B-19_marine_sediment_group*. *Rhodopirellula* is a unique indicator genus of BL. Indicator species belong to the CV community, including *Pir4_lineage, Pseudomonas, Acinetobacter, Blastocatella, Stenotrophobacter*, and *Flavisolibacter*. LC has its own indicators: *Pirellula, RB41, Streptomyces, AKYG587*, and *Candidatus_Udaeobacter*. *Ralstonia, Bryobacter, Sphingomonas, Flavobacterium, Nitrospira, Terrimonas*, and *Subgroup_10* are indicator species belonging to the PA plant community. *Truepera, Anditalea, Pontibacter, Ilumatobacter, Azoarcus, Bacillus*, and *Luteolibacter* are the indicator species of PC. The abundant fungi come from Ascomycota, and the indicator species of the BL community include *Cercospora, Ascochyta, Cunninghamella, Candida*, and *Aspergillus*. *Periconia* is not an indicator species; the rest are indicator species of different plant communities.

**Figure 4:**
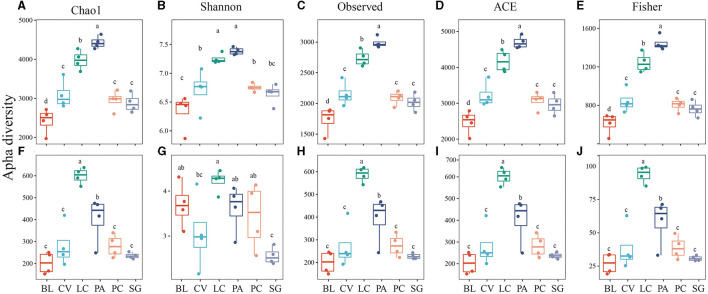
Alpha diversity of bacteria **(A–E)** and fungi **(F–J)** in different plant communities.

**Figure 5:**
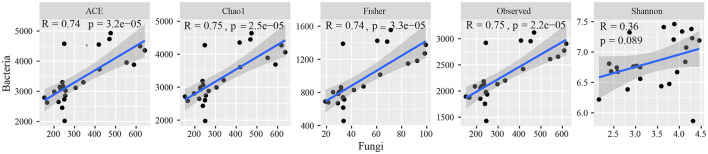
Relationships of bacterial alpha diversity to fungal diversity across different plant communities.

In the soda-saline environment (Figure 4), among the bacteria, the diversity of PA was the highest, followed by LC, which was higher than that in other communities. In fungi, the diversity of LC was the highest, and the Shannon index of SG was significantly lower than that of the BL plant community. In addition, the observed species of the phylogenetic bacterial community of ASV, Fisher, Chao1, and ACE were positively correlated with the fungal community (Figure 5).

**Figure 6:**
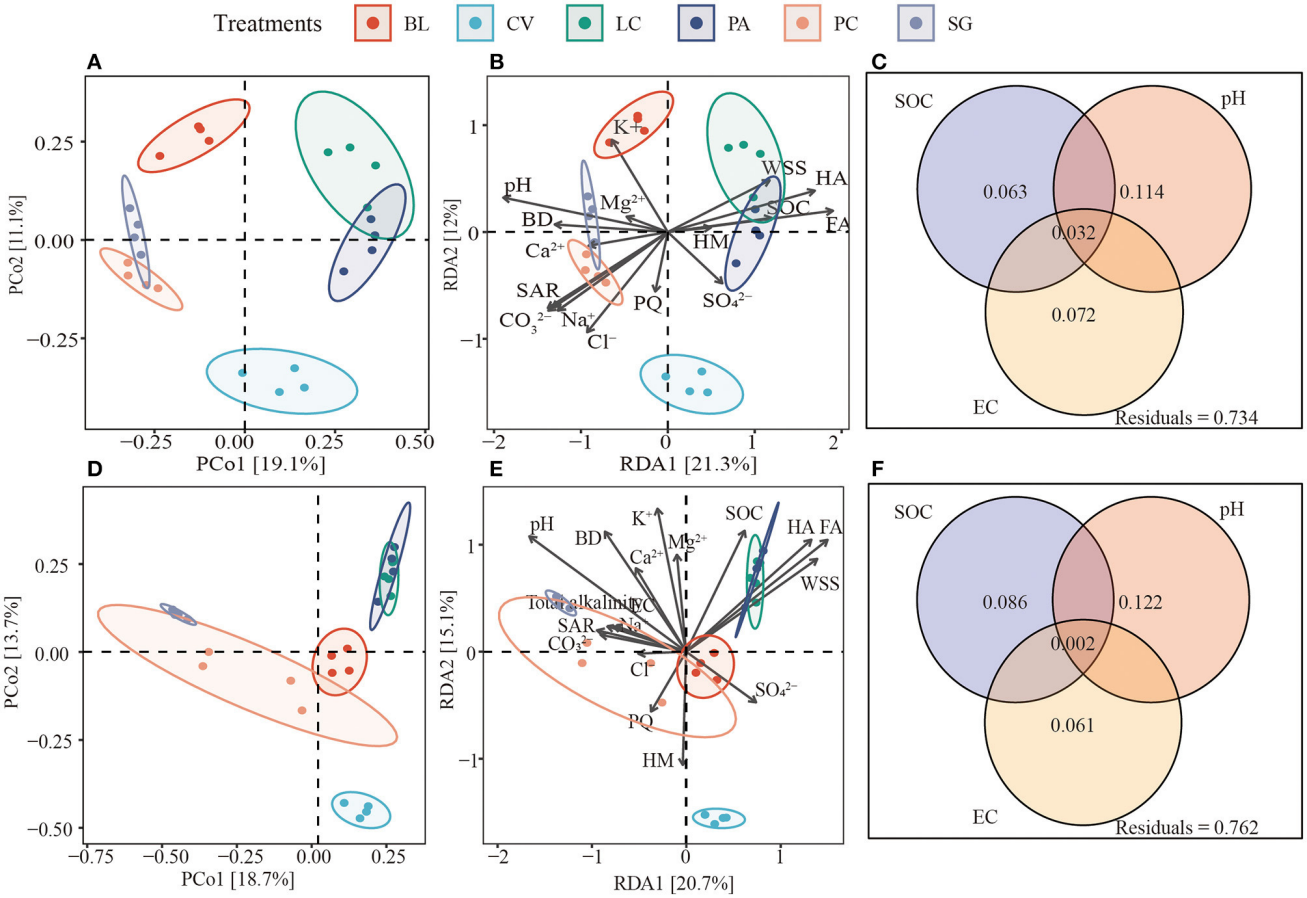
Differences in soil bacterial and fungal communities **(A, D)**. Redundancy analysis **(B, E)** shows bacterial and fungal communities and environmental factors between soils of different plant communities. **(C, F)** The contribution of soil organic matter (SOC), soil pH, and soil electrical conductivity (EC) to the composition of bacterial and fungal communities based on variance distribution analysis. BL, bare land without vegetation on the surface; CV, *Chloris virgata*; LC, *Leymus chinensis*; PA, *Phragmites australis*; PC, *Puccinellia chinampoensis*; SG, *Suaeda glauca*; WSS, water-soluble organic matter; FA, fulvic acid; HA, humic acid; HM, humin; SOC, soil organic carbon; EC, electrical conductivity; BD, bulk density; and SAR, sodium adsorption ratio.

PCoA plots based on Bray-Curtis distances clearly showed that there were significant differences between fungal and bacterial soil microbial communities (Supplementary Table S2). In the case of bacteria, environmental factors explained 33.3% of the community variation, and for fungi, environmental factors explained 35.8% of the community variation. Variations in bacterial and fungal communities were mainly explained by pH. The SOC explained more variation for fungi (0.086 vs. 0.063) than bacteria (Figure 6).

### 3.3. Factors affecting soil organic matter in different plant communities

**Figure 7:**
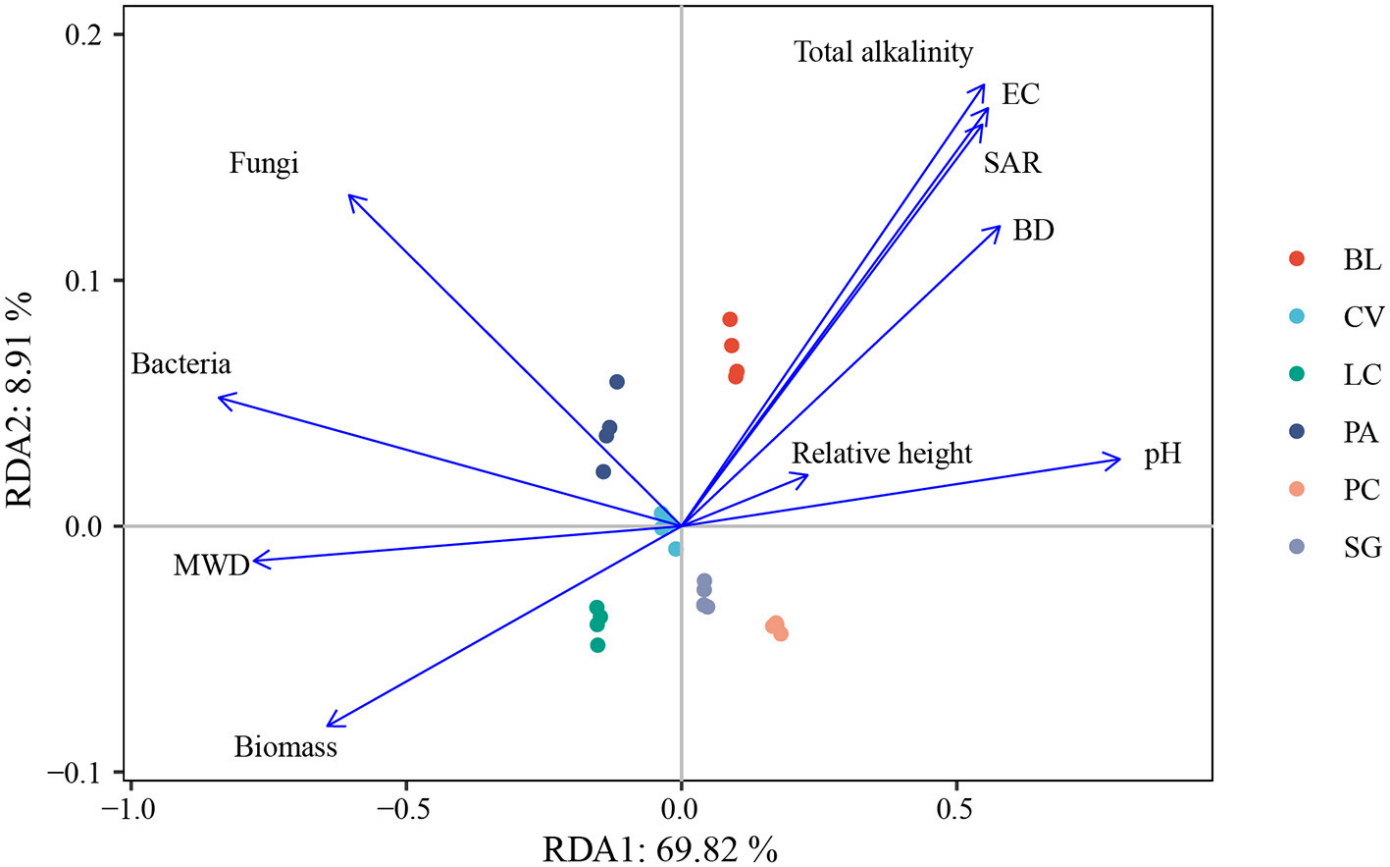
Ordination plots of redundancy analysis to identify the relationships among soil bacteria, fungal communities, environmental factors, and soil humus. BL, bare land without vegetation on the surface; CV, *Chloris virgata*; LC, *Leymus chinensis*; PA, *Phragmites australis*; PC, *Puccinellia chinampoensis*; SG, *Suaeda glauca*; EC, electrical conductivity; BD, bulk density; SAR, sodium adsorption ratio; and MWD, mean weight diameter.

Pearson correlation analysis showed that Actinobacteria, Gemmatimonadetes, and Ascomycota were significantly negatively correlated with FA, HA, and SOC. Acidobacteria and Mortierellomycota were significantly positively correlated with WSS, FA, HA, and SOC (*p* < 0.05). Gemmatimonadetes and Ascomycota were significantly correlated with WSS (Table 2). Redundancy analysis showed that soil environmental factors and microorganisms accounted for 78.34% of the total variation in SOM components (Figure 7), and bacterial communities, pH, MWD, and fungi communities were the main factors affecting SOM components (Supplementary Figure S2).

**Table 2:** Correlations between soil microbial communities and soil humus.

	**WSS (g/kg)**	**FA (g/kg)**	**HA (g/kg)**	**HM (g/kg)**	**SOC (g/kg)**
Proteobacteria	0.280	0.247	0.104	−0.088	0.016
Actinobacteria	−0.292	−0.689^**^	−0.560^**^	−0.334	−0.520^**^
Bacteroidetes	0.061	−0.220	−0.262	−0.248	−0.249
Acidobacteria	0.474^*^	0.863^**^	0.760^**^	0.391	0.628^**^
Gemmatimonadetes	−0.441^*^	−0.664^**^	−0.601^**^	−0.182	−0.407^*^
Planctomycetes	−0.376	−0.065	0.031	0.075	0.054
Firmicutes	−0.273	−0.088	0.034	0.309	0.182
Chloroflexi	0.162	−0.390	−0.420^*^	−0.660^**^	−0.603^**^
Ascomycota	−0.482^*^	−0.564^**^	−0.504^*^	−0.279	−0.413^*^
Mortierellomycota	0.661^**^	0.835^**^	0.726^**^	0.374	0.605^**^
Basidiomycota	−0.320	−0.370	−0.313	0.029	−0.145

^*^ and ^**^ indicate significance at 0.01 <*p* < 0.05 and 0.001 <*p* < 0.01 respectively.

WSS, water-soluble organic matter; FA, fulvic acid; HA, humic acid; HM, humin; and SOC, soil organic carbon.

## 4. Discussion

Studies have shown that vegetation has a certain resistance to soil salinity (Negrão et al., 2016; Ma et al., 2020; Han et al., 2021). Bare land has the highest soil salinity. Among vegetation types, the reduction of soil salinity was most obvious in PA and LC, which can be attributed to the elevated organic matter content and MWD of PA and LC. These factors facilitate microorganisms' enhanced diversity and activity, leading to the dissolution of soil CaCO_3_.

As a result, Na^+^ ions adsorbed on the surface of soda-saline soil are effectively replaced by Ca^2+^ ions, expediting the desalination process and improving the physical and chemical properties of the soil (Qadir et al., 2007). Owing to the high pH of the soil and its high electrical conductivity, the rate of soil humification was affected, the accumulation of humus was reduced, and the conversion rate of FA to HA in the soil was also slow, resulting in a low PQ value (Zhao et al., 2019). With the regeneration of LC communities, biomass increases, which strengthens the arrangement of soil particles, further promotes the assembly of microaggregates into macroaggregates, and enhances soil structural stability with higher MWD, which helps keep organic matter from erosion and decomposition (Zhao et al., 2018; Feng et al., 2021). In our study, SOCs of different vegetation communities showed an obvious increasing trend from bare land to squares covered by adapted vegetation. Regarding the increase in soil HA and FA contents with increasing SOC content, the increase in SOC content under different vegetation types was also accompanied by higher MWD and higher proportions of HA and FA in SOM, indicating improved soil structure and humus quality. For different vegetation types, soil salinity and soil organic carbon content showed a significant negative correlation, and salinity had a small correlation with soil PQ value (Supplementary Figure S1). This showed that soil salinity under different plant communities affected the total soil organic carbon content. In the case of high salinity, soil organic carbon content was low. High salinity affects the growth of plants and reduces the return of organic residues to the soil, resulting in a decrease in soil organic matter.

Spontaneous vegetation regeneration significantly influences the diversity or richness of soil microbial communities (Liu et al., 2021). Chang et al. (2022) sequenced soil bacterial communities under different vegetation conditions and found that vegetation coverage can significantly increase bacterial ACE, Chao1, Shannon, and Simpson index communities. This is consistent with our findings that plant community reestablishment can enhance soil microbial diversity. Our results showed that Proteus, Acidobacteria, Actinomycetes, Bacteroidetes, and Monoas are the main bacterial species at all examined vegetated sites. In communities with higher SOC, such as LC and PA, the abundance of the Acidobacteria phylum is significantly increased. In addition, the abundance of the Actinomycetes phylum decreased, which is consistent with findings from previous studies and shows that these phyla are common and ubiquitous in soil (Dai et al., 2017). Acidobacteria harbor a broad library of carbohydrate-active enzymes that encode a wide range of carbohydrates and are involved in the breakdown, utilization, and biosynthesis of a wide variety of carbohydrates (Dedysh and Damsté, 2018). Acidobacteria play a beneficial role by selectively regulating the ecological processes of the host ecosystem. These bacteria provide two important ecological services, namely SOM decomposition and denitrification, to enhance carbon stability (Banerjee et al., 2018). These bacteria may enhance the key ecological process of soil carbon fixation. As members of Acidobacteria have been identified as the structural and functional cornerstone of the plant-soil microbiome and agricultural ecosystem (Kalam et al., 2020), studies have shown that Acidobacteria have a positive effect on plant root growth (da Rocha et al., 2010). Therefore, their existence can improve the performance and productivity of plants. Actinomycetes are K-strategy microorganisms resistant to stress (Bérard et al., 2015).

Most of the fungi belonged to Ascomycota. SG and CV significantly increased the abundance of Ascomycota. PA and LC plant communities increased the abundance of Mortierellomycota. BL significantly increased the relative abundance of Mucoromycota. It was reported in the literature that Ascomycetes were not adapted to grow in high-nutrient environments (Wu et al., 2019). Therefore, the increase in the relative abundance of Ascomycetes resulted from the higher salinity and lower organic matter content of SG and CV plant communities.

According to the redundancy analysis, the content and composition of soil organic matter varied significantly among different plant communities, which was mainly related to bacterial communities, pH, MWD, and fungal communities (Supplementary Figure S2). Among these factors, pH was the most important factor causing the difference in microbial communities (Figure 5). As the plant residues enter the soil, the differences in the microbial communities of plant communities lead to differences in the nature of soil organic matter produced by the decomposition of the residues (Kallenbach et al., 2016; Domeignoz-Horta et al., 2021). LC and PA plant communities can reduce soil pH, cause soil particles to aggregate (Wong et al., 2010), improve soil aggregation, protect soil organic matter from decomposition (Zhang et al., 2021), and change the soil microbial community, increasing soil organic matter content. This indicates that soil pH can indirectly affect the content and composition of soil organic matter by regulating soil microbial communities and soil aggregation ability, which shows that reducing soil pH is a key step in the process of restoring vegetation in soda-saline soil to increase soil carbon sequestration and fertility in the future.

## 5. Conclusion

During our soda-saline vegetation restoration tests, distinct plant communities exhibited the capacity to elevate SOC content and modify its composition compared to bare land. Concurrently, there was a noticeable enhancement in soil aggregation, bolstering plant productivity. Furthermore, these findings underscore the pivotal role of soil microbial communities in orchestrating organic matter alterations. An affirmative correlation was established between SOC content, Acidobacteria, and Mortierella, while Actinobacteria, Gemmatimonadetes, Ascomycota, and SOC content displayed significant negative correlations. Notably, pH emerged as a potent explanatory factor, accounting for 11.4% and 12.2% of the variability observed in soil bacteria and fungi, respectively. Hence, pH emerged as the primary driver behind microbial transformations. The observed disparities in SOC composition and content within soda-saline vegetation can primarily be attributed to variations in pH levels.

Consequently, reducing soil pH emerges as a pivotal step in the prospective process of vegetation restoration in soda-saline environments. The prohibition of grazing practices holds the potential to elevate soda-saline SOC content and foster microbial diversity. Among the spectrum of different vegetation types, *Leymus chinensis* and *Phragmites australis* stand out due to their heightened SOC carbon content and microbial diversity.

## Data availability statement

The data presented in the study are deposited in the NCBI repository, accession number PRJNA935485 and PRJNA935506.

## Author contributions

FY and LG conceived the research. LG performed the data analysis and wrote the manuscript. FY, ZW, and TT edited the manuscript. All authors reviewed and approved the manuscript, contributed to the article, and approved the submitted version.
