# Supplementary material for: Effects of different types of vegetation cover on soil microorganisms and humus characteristics of soda-saline land in the Songnen Plain
Source: Front Microbiol. 2023 Sep 21;14:1163444. doi: 10.3389/fmicb.2023.1163444 (PMC10551454; doi:10.3389/fmicb.2023.1163444)
Supplement: Supplementary file 1 [file Data_Sheet_1.docx]

Table S1 Vegetation characteristics of sampled squares

|  | Relative height(m) | Dominant plant species | Plant cover(%) | Average plant height (cm) | Biomass（g/m2） | Other plant species |
| --- | --- | --- | --- | --- | --- | --- |
| BL | 0.44 |  | 0.00 | 0.00 | 0.00 |  |
| BL | 0.61 |  | 0.00 | 0.00 | 0.00 |  |
| BL | 0.54 |  | 0.00 | 0.00 | 0.00 |  |
| BL | 0.71 |  | 0.00 | 0.00 | 0.00 |  |
| CV | 0.50 | *Chloris virgata* | 85.00 | 41.50 | 193.44 | *Lespedeza daurica*, *Artemisia scoparia* |
| CV | 0.52 | *Chloris virgata* | 72.00 | 11.67 | 118.72 | *Artemisia scoparia* |
| CV | 0.43 | *Chloris virgata* | 75.00 | 18.17 | 82.24 | *Artemisia scoparia* |
| CV | 0.49 | *Chloris virgata* | 80.00 | 13.50 | 112.48 | *Artemisia scoparia* |
| LC | 0.54 | *Leymus chinensis* | 85.00 | 54.00 | 329.13 | *Artemisia scoparia*, *Puccinellia chinampoensis* |
| LC | 0.46 | *Leymus chinensis* | 88.00 | 56.00 | 244.04 | *Artemisia scoparia*, *Artemisia sieversiana* |
| LC | 0.46 | *Leymus chinensis* | 98.00 | 65.50 | 377.48 | *Puccinellia chinampoensis*, *Saussurea runcinata* |
| LC | 0.36 | *Leymus chinensis* | 70.00 | 51.17 | 463.24 | *Artemisia scoparia*, *Saussurea runcinata* |
| PA | 0.14 | *Phragmites australis* | 75.00 | 104.00 | 237.15 | *Lathyrus quinquenervius* |
| PA | 0.10 | *Phragmites australis* | 96.00 | 144.17 | 489.08 | *Cirsium setosum*, *Cynanchum chinense* |
| PA | 0.08 | *Phragmites australis* | 80.00 | 104.67 | 432.52 | *Suaeda glauca* |
| PA | 0.16 | *Phragmites australis* | 93.00 | 109.33 | 485.00 | *Artemisia sieversiana* |
| PC | 0.47 | *Puccinellia chinampoensis* | 70.00 | 71.33 | 168.79 | *Suaeda glauca* |
| PC | 0.33 | *Puccinellia chinampoensis* | 60.00 | 83.50 | 163.00 |  |
| PC | 0.29 | *Puccinellia chinampoensis* | 65.00 | 75.00 | 243.92 | *Artemisia scoparia*, *Sonchus wightianus* |
| PC | 0.08 | *Puccinellia chinampoensis* | 70.00 | 76.50 | 158.64 | *Polygonum lapathifolium* |
| SG | 0.38 | *Suaeda glauca* | 55.00 | 4.50 | 64.64 | *Artemisia scoparia* |
| SG | 0.41 | *Suaeda glauca* | 65.00 | 6.67 | 108.72 |  |
| SG | 0.41 | *Suaeda glauca* | 53.00 | 5.60 | 75.00 | *Artemisia scoparia* |
| SG | 0.45 | *Suaeda glauca* | 50.00 | 5.83 | 90.84 |  |

Note: BL: bare land without vegetation on the surface; CV: *Chloris virgata*; LC: *Leymus chinensis*; PA: *Phragmites australis*; PC: *Puccinellia chinampoensis*; SG: *Suaeda glauca*.

Table S2 The permutational multivariate analysis of variance (PERMANOVA) based on Bray-Curtis distance among different plant communities.

| Comparison | Bacteria | | | Fungi | | | |
| --- | --- | --- | --- | --- | --- | --- | --- |
|  | F | R^2^ | P | F | R^2^ | | P |
| BL vs CV | 3.873097 | 0.392288 | 0.032 | 3.085259 | | 0.33959 | 0.022 |
| BL vs LC | 3.557951 | 0.37225 | 0.037 | 2.727757 | | 0.312538 | 0.034 |
| BL vs PA | 4.039587 | 0.402366 | 0.03 | 2.72393 | | 0.312237 | 0.037 |
| BL vs PC | 3.983248 | 0.398993 | 0.03 | 2.245139 | | 0.272299 | 0.028 |
| BL vs SG | 3.827612 | 0.389475 | 0.031 | 4.085379 | | 0.405079 | 0.038 |
| CV vs LC | 3.048334 | 0.336895 | 0.029 | 5.012598 | | 0.455169 | 0.029 |
| CV vs PA | 2.752772 | 0.314503 | 0.029 | 5.017412 | | 0.455408 | 0.031 |
| CV vs PC | 3.615878 | 0.376032 | 0.023 | 3.436271 | | 0.364156 | 0.035 |
| CV vs SG | 4.149176 | 0.408819 | 0.02 | 8.275907 | | 0.579712 | 0.03 |
| LC vs PA | 1.978005 | 0.247932 | 0.03 | 3.292048 | | 0.354287 | 0.031 |
| LC vs PC | 4.374511 | 0.42166 | 0.025 | 3.666084 | | 0.379273 | 0.023 |
| LC vs SG | 4.105961 | 0.406291 | 0.035 | 6.801781 | | 0.531315 | 0.022 |
| PA vs PC | 4.501099 | 0.428631 | 0.029 | 3.597622 | | 0.374845 | 0.03 |
| PA vs SG | 4.414642 | 0.423888 | 0.028 | 6.800313 | | 0.531262 | 0.022 |
| PC vs SG | 2.5232 | 0.296039 | 0.032 | 2.483826 | | 0.292772 | 0.038 |

Note: BL: bare land without vegetation on the surface; CV: *Chloris virgata*; LC: *Leymus chinensis*; PA: *Phragmites australis*; PC: *Puccinellia chinampoensis*; SG: *Suaeda glauca*.


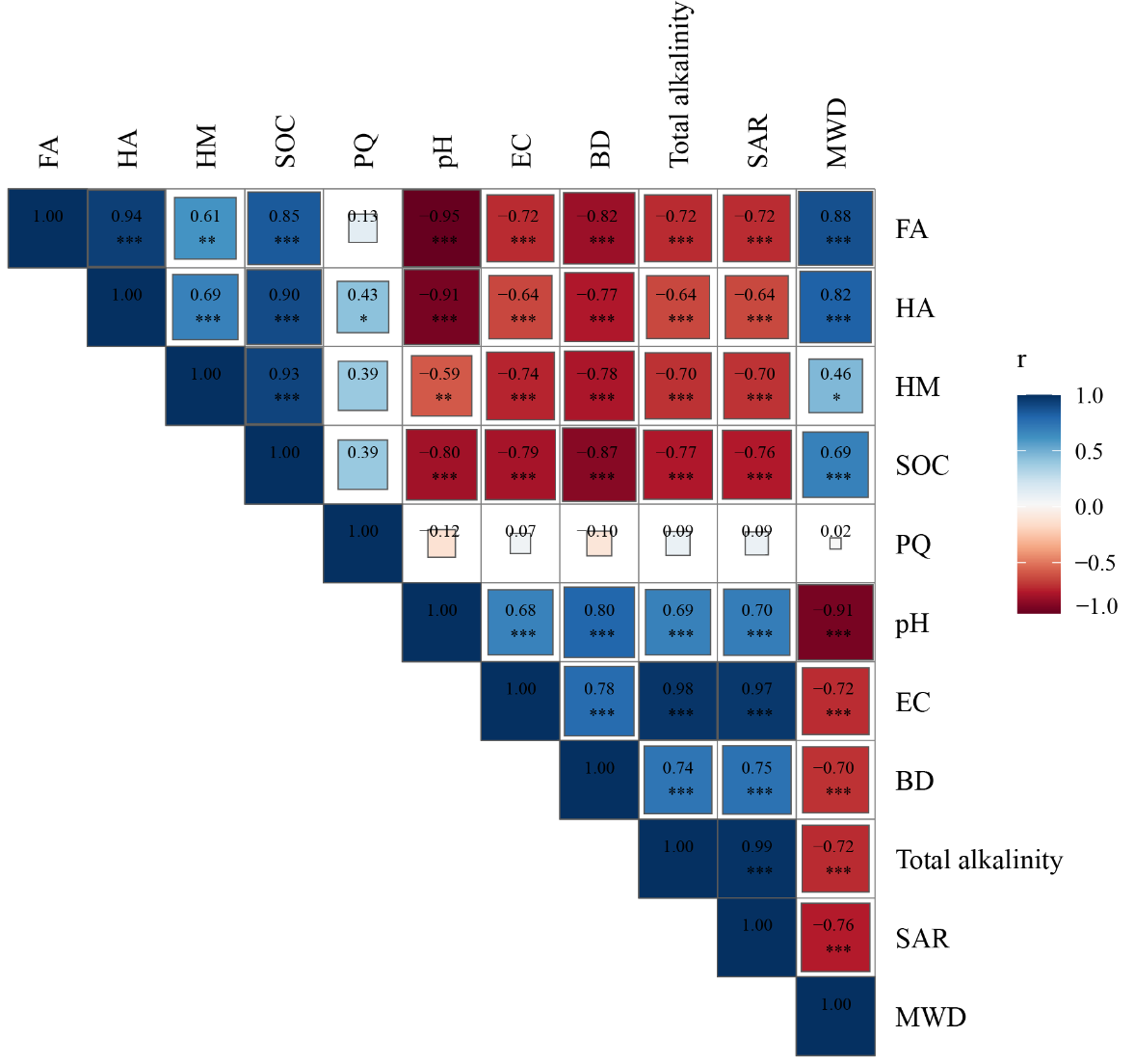
Figure1S Pearson correlation of soil physical and chemical properties. *, ** and *** indicate significance at 0.01 < p < 0.05, 0.001 < p < 0.01 and p < 0.001, respectively. WSS: water extracted organic matter; FA: fulvic acid; HA: humic acid; HM: humin; SOC: soil organic carbon; EC: electrical conductivity; BD: bulk density; SAR: sodium adsorption ratio; MWD: mean weight diameter.


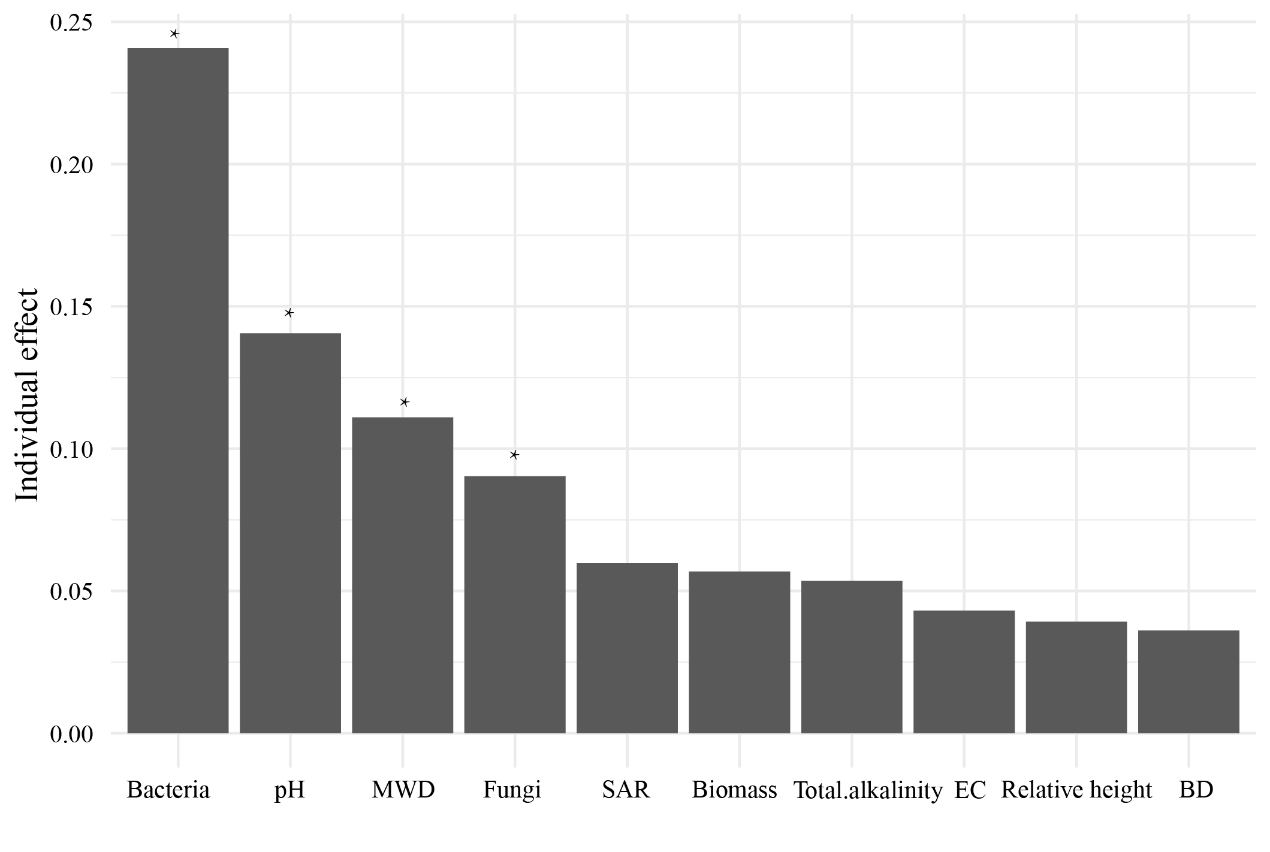
Figure S2 Impact of soil bacterial, fungi communities and environmental factors on the rate of soil humus. EC: electrical conductivity; BD: bulk density; SAR: sodium adsorption ratio; MWD: mean weight diameter. * indicate significance at 0.01 < p < 0.05.
